# The Problem of Thresholding in Small-World Network Analysis

**DOI:** 10.1371/journal.pone.0053199

**Published:** 2013-01-03

**Authors:** Nicolas Langer, Andreas Pedroni, Lutz Jäncke

**Affiliations:** 1 Division Neuropsychology, Institute of Psychology, University of Zurich, Zurich, Switzerland; 2 Laboratories of Cognitive Neuroscience, Division of Developmental Medicine, Department of Medicine, Children’s Hospital, Boston, Massachusetts, United States of America; 3 Harvard Medical School, Boston, Massachusetts, United States of America; 4 Division Social and Affective Neuroscience, Department of Psychology, University of Basel, Basel, Switzerland; 5 Center for Economic Psychology, Department of Psychology, University of Basel, Basel, Switzerland; 6 The International Normal Aging and Plasticity Imaging Center, Zurich, Switzerland; University of Michigan, United States of America

## Abstract

Graph theory deterministically models networks as sets of vertices, which are linked by connections. Such mathematical representation of networks, called graphs are increasingly used in neuroscience to model functional brain networks. It was shown that many forms of structural and functional brain networks have small-world characteristics, thus, constitute networks of dense local and highly effective distal information processing. Motivated by a previous small-world connectivity analysis of resting EEG-data we explored implications of a commonly used analysis approach. This common course of analysis is to compare small-world characteristics between two groups using classical inferential statistics. This however, becomes problematic when using measures of inter-subject correlations, as it is the case in commonly used brain imaging methods such as structural and diffusion tensor imaging with the exception of fibre tracking. Since for each voxel, or region there is only one data point, a measure of connectivity can only be computed for a group. To empirically determine an adequate small-world network threshold and to generate the necessary distribution of measures for classical inferential statistics, samples are generated by thresholding the networks on the group level over a range of thresholds. We believe that there are mainly two problems with this approach. First, the number of thresholded networks is arbitrary. Second, the obtained thresholded networks are not independent samples. Both issues become problematic when using commonly applied parametric statistical tests. Here, we demonstrate potential consequences of the number of thresholds and non-independency of samples in two examples (using artificial data and EEG data). Consequently alternative approaches are presented, which overcome these methodological issues.

## Introduction

The human brain is organized as a highly interconnected structural network that functionally connects adjacent and distant brain areas [1]. In the last decade, there’s an increasing interest in modeling the human brain network using brain graphs, because they seem to provide an adequate, yet simple model of a complex system as the brain is. A brain graph models the connectivity of the brain with a number of nodes interconnected by a set of edges [2]. The constitution of a node within a brain graph has to be specified by the researcher and is depending on neuroimaging method, anatomical parcellation schemes and connectivity measures [3]. Moreover, one edge of such a brain graph can represent a functional or structural connection between cortical or subcortical regional nodes. Such a network can be mathematically represented as a graph with edges and nodes. The resulting topology is characterized by local and global parameters, most prominently, the cliquishness of connections between nodes in a topological neighbourhood of the graph (clustering coefficient), or the global efficiency of information transfer within the network, which refers to the path length of a network [2], [4].

Networks of so-called “small-world” topology constitute an ideal balance of efficient information transmissions between distant nodes (small path length), while retaining efficient local information processing (high clustering coefficient) [2], [5]. These premises lead to a topology characterized by segregated clusters that are connected by local hubs, suggesting functional integration and segregation, which is a highly plausible model of how the human brain operates. This view is supported by studies indicating that brain networks at the scale of single neurons up to macroscopic functional networks incorporate the topology of such “small-worldness” [1], [2], [3]. Interestingly, a growing number of studies indicates that small-world characteristics based on anatomical and functional brain measures are strongly related to intelligence [6], [7], [8], age [9], [10], sex [11], genetics [12], synaesthesia [13], and/or neurological diseases [14], [15], [16], [17]. Thereby, indicating that this network topology is a key factor in describing brain functions.

Although this research strategy provides promising insights, the commonly used analysis approach is associated with some particular statistical problems. In this paper we will discuss these problems and will present two alternative approaches that overcome these methodological issues.

Usually, small-world network analyses in the context of exploring interindividal differences aim to test whether parameters of network efficiency (i.e. path length and average cluster coefficient) are related to specific populations. For example, the researcher aims to examine whether two groups differ in terms of particular network parameters. In order to accomplish this comparison, the network parameters are calculated for each group separately and then compared between these groups using parametric tests, such as, t-tests or ANOVAs. A common approach is to calculate various measures of dependency (i.e. correlation) between brain attributes obtained from regions of interest (i.e. cortical thickness, brain activity, etc.) that are extracted from anatomical or neurophysiological data (i.e. EEG, MEG. fMRI, MRI, or DTI). This leads to regions-wise within-subject measures of connectivity. If measures of connectivity are obtained for each group separately - as with structural magnetic resonance imaging (sMRI) and diffusion tensor imaging (DTI) data (except for fibre tracking data) - [14], [18] only one network per group and per threshold can be calculated, leading to region-wise within-group measures of connectivity.

A commonly used strategy to conduct statistical comparisons for the latter type of data is to use different and arbitrarily chosen thresholds from which the different network measures are calculated [1], [2], [3]. As a consequence of this strategy one obtains as many network measures per group as thresholds used. These different thresholded networks are pseudo-replications of group level networks, which serve as measures for classical inferential statistics. In the context of this paper we will use the expression “multiple-thresholds-approach” to describe this analysis procedure.

Although frequently used, this “multiple-thresholds-approach” is associated with several problems. First, depending on the number of chosen thresholds the sample size will vary and this influences the power of statistical testing. Second, the sets of thresholded mean correlation matrices are not independent (as classical statistics would require), because the information in a sparser correlation matrix is also comprised in a denser correlation matrix. This is particularly problematic for parametric statistical tests, since they inevitably require independence of the data. Thirdly, not only the number of thresholds used causes problems, but also the range of the thresholds used to estimate the network parameters are arbitrary. For example, one could restrict the thresholds to a range from 0.2 to 0.6 or to a range from 0.3 to 0.8. Using these different ranges will generate different results.

Although the above-mentioned approach is not entirely wrong, since one may wish to compare the profiles of network parameters across the different thresholds, this approach can nevertheless lead to ambiguous results. In this paper, we will demonstrate with two examples how this approach can lead to ambiguous results. In the last part we will propose an alternative approach, which uses randomisation statistics and does not suffer from the above-mentioned statistical problems.

## Methods

### Ethics Statement

This study was conducted according to the principles expressed in the Declaration of Helsinki. The study was approved by the local ethics committee (Kantonale Ethikkomission: EK-80/2008). All participants provided written informed consent for the collection of samples and subsequent analysis.

### Multiple-thresholds-approach

#### Example 1 - real data

For the first illustration of the problem associated with this approach we used EEG data from a previous study [19]. Seventy-four healthy male students (mean/standard deviation: 25,5/4.86 years) participated in the study. After recording seven minutes of spontaneous EEG at rest, subjects conducted the Raven Advanced Progressive Matrices (RAPM) [20], which is a widely used measure of psychometric intelligence. In contrast to the previous study [19], we performed a median-split based on the performance in the RAPM. This resulted in a high IQ group (n  = 25) and a low IQ group (n  = 34). The median raw score was 23 correctly solved items. Subjects who scored at the median level of the RAPM were excluded. Spontaneous EEG at rest was used to analyze connectivity parameters of intracortical sources of brain oscillations in the upper alpha band (10,5–12 Hz). The coherence between 84 anatomical regions of interest in both hemispheres was computed (for the details of the analyses see [19]). This resulted in an 84×84 correlation matrix (84 ROIs) for each subject. The connectivity matrices of all subjects from the low IQ group to the high IQ group were averaged separately, resulting in a mean connectivity matrix for the low IQ and high IQ groups. The connectivity matrices were then thresholded at different coherence values. This multiple-threshold approach resulted in as many networks per group as the number of thresholds applied to the connectivity matrix. Network parameters (clustering coefficient, characteristic path length and number of edges) were then calculated for each connectivity matrix by using the tnet software [21]. In order to draw statistical inferences regarding group differences in network parameters, such as, the clustering coefficient and the characteristic path length, we used a classical parametric statistical test (t-test for independent samples). As mentioned above, this multiple-threshold-approach is problematic because both the sample size and the statistical power depend on the number of thresholds used. In addition, the key assumption of independency between samples in t-tests is violated when using differently thresholded correlation matrices.

We demonstrated this by using three different numbers of thresholds while keeping the ranges constant (range: 0.65–0.99). The sparsest network (threshold r  = 0.99) was omitted, because the networks became no longer consistent. In the first trial we thresholded the connectivity matrix 10 times (increments: 0.034) resulting in 10 networks per group, in the second trial we thresholded the connectivity matrix 15 times (increments: 0.0227), and in the third trial we thresholded the connectivity matrix 35 times (increments 0.01). In a second step, the small-world parameters were calculated for each threshold per group. The different thresholded networks served as the different measurements units within each group.

Thus, in the first trial we obtained 10 measurements for each small-world parameter, in the second trial we obtained 15 measurements for each small-world parameter, and in the third trial we obtained 35 measurements for each small-world parameter. Afterwards, we separately compared these small-world parameters between the low IQ and the high IQ groups for each trial by using a t-test for independent samples (p<0.05). Since we have to consider the fact that p-values depend on sample size, we also calculated effect sizes according to Cohen [22]. All statistical analyses in the present study were performed with MATLAB [23].

#### Results

**Figure 1:**
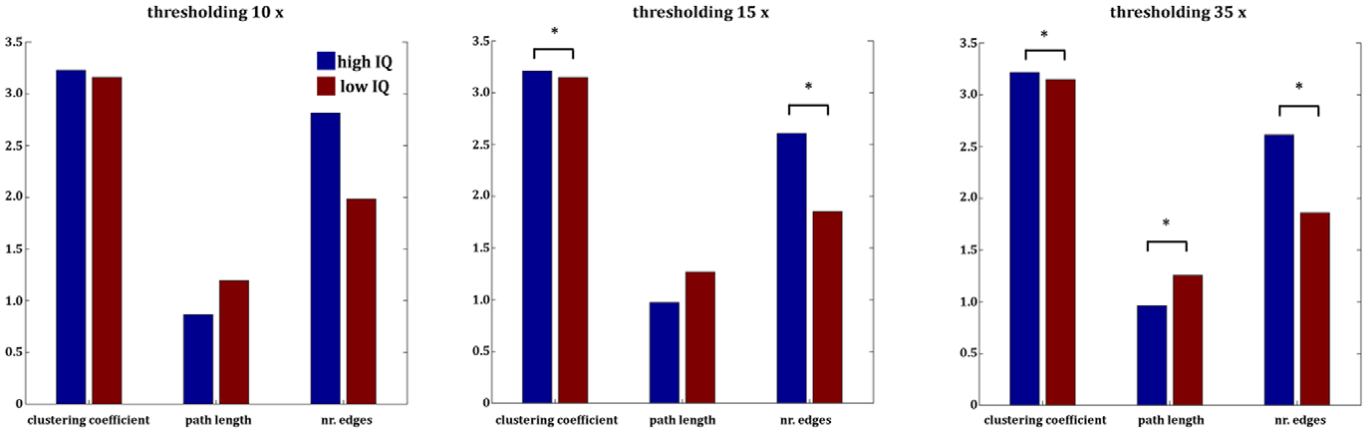
Results of the *multiple-thresholds-approach* of the example with the real data. Mean values for the small-world parameters clustering coefficient, path length, and number of edges. We thresholded the correlation matrix 10, 15, and 35 times; this resulted in different statistical results. For the version with 10 increments, t-tests revealed no statistical differences. For the version with 15 increments, the clustering coefficient and number of edges was significantly increased in the high IQ group compared to the low IQ group. In the version with 35 different thresholds, the comparison between the high and low IQ groups revealed significant effects for all small-world parameters. The high IQ group showed a significantly enhanced small-world topology. For an optimized display, the numbers of edges were scaled (number of edges divided by 1000).

For the first trial (thresholding the matrix 10 times), there were no significant differences between the low and the high IQ groups regarding small-world parameters (clustering coefficient: t_(8)_  = 1.87, p  = 0.078, Cohen’s d  = 0.42; path length: t_(8)_ = −1.30, p  = 0.21, Cohen’s d  = 0.31; number of edges: t_(8)_  = 1.85, p  = 0.08, Cohen’s d  = 0.42). For the second trial (thresholding the matrix 15 times), we found significantly more edges (t_(13)_  = 2.40, p  = 0.02, Cohen’s d  = 0.38), a higher cluster coefficient (t_(13)_  = 3.07, p  = 0.004, Cohen’s d  = 0.46), and no differences regarding characteristic path length (t_(13)_ = −1.51, p  = 0.14, Cohen’s d  = 0.25) for the high IQ group compared to the low IQ group. For the third trial (thresholding the matrix 35 times), t-tests revealed highly significant differences between the high and the low IQ groups. There was a significantly increased number of edges (t_(33)_  = 3.52, p  = 7.76*10^−4^, Cohen’s d  = 0.39), and a higher clustering coefficient (t_(33)_  = 4.44, p  = 3.33*10^−5^, Cohen’s d  = 0.47) in the high IQ group. In contrast, we found a significantly decreased characteristic path length (t_(33)_ = −2.24, p  = 0.02, Cohen’s d  = 0.26). An overview of this data is presented in Figure 1.

#### Example 2– simulated data

**Figure 2:**
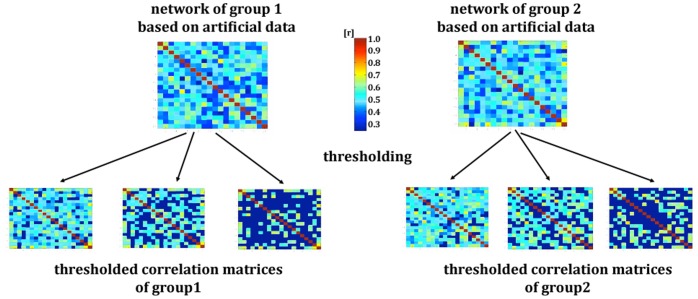
Procedure of the *multiple-thresholds-approach* with artificial data. Networks of two groups based on artificial data. The networks were thresholded over a set of thresholds.

In our second example, we use a simulation to illustrate how the commonly used multiple-threshold-approach may lead to false positive results. An illustration of the method is displayed in Figure 2. We set up our simulation to mimic the multiple-threshold-approach with data obtained by structural MRI or FA-DTI data. We simulated a study with 60 subjects, who comprised two experimental groups of equal size (30 subjects per group). This is a commonly used sample size for studies conducted in this field [2], [24], [25]. As in the first example, we used 84 brain regions (e.g. 84 Brodmann Areas). A randomly created value of a z-distribution was allocated for each of the 84 brain regions. This was done separately for each subject. Since we only have one value per node and sample, there is no possibility of calculating a correlation matrix for a single subject. Therefore, in order to calculate the strength of the association between nodes, we needed to calculate correlations between the nodes (84 brain regions) of each group. This results in two association matrices with 84 rows and columns. Each entry of the row and column represents the correlation coefficient (connectivity strength) between the two simulated brain regions. Since there is now only one network per group, the groups cannot be statistically compared at this stage. We followed the common multiple-threshold-approach to “deal” with this problem by thresholding the two networks over a set of thresholds (range: 0.01–0.91; increments: 0.001, total: 900); this resulted in 900 networks per group. For each thresholded network we then obtained the small-world parameters, namely, the number of edges, the clustering coefficient, and the characteristic path length by using the tnet software [21]. To compare the small-world network parameters, t-tests for independent samples (p<0.05) were used, which is common practice. We calculated three examples (three different threshold ranges) with the simulation data because we aimed to replicate the analysis and to demonstrate that in addition to the number of thresholds, the threshold limits (upper and lower threshold of the threshold range) might influence the results. In the first step, we extracted three different threshold ranges between 0.01 and 0.91. A low threshold range (0.01–0.06), a middle threshold range (0.50–0.54), and a high threshold range (0.86–0.91) were chosen. This resulted in 50 differently thresholded connectivity matrices per group within the threshold range. Analog to the example of the real data, we compared the networks of the two simulated groups over different numbers of thresholds. The different thresholded connectivity matrices served as the different measurement units within each group. In the first trial, we took 10 differently thresholded connectivity matrices (increments: 0.005) for the group comparison using independent t-tests. In the second trial, we calculated with 25 connectivity matrices (increments: 0.002) per group. In the third trial, we calculated with 50 connectivity matrices (increments: 0.001) per group. This was done for the three threshold ranges (0.01–0.06; 0.50–0.54; 0.86–0.91). Because the networks were randomly generated, we hypothesized that there would be no differences between the networks of the two groups in any small-world parameter.

#### Results

Comparing the random networks of the two simulated groups for the first trial (10 thresholded connectivity matrices) within the threshold range of 0.86–0.91 revealed no significant difference in any of the small-world parameters. In the second trial (25 thresholded connectivity matrices), we found significantly more edges (t_(23)_  = 2.18, p,  = 0.03, Cohen’s d  = 0.29) and a lower characteristic path length (t_(23)_ = −2.09, p,  = 0.04, Cohen’s d  = 0.28) for the first group. For the third trial (50 thresholded connectivity matrices), the t-tests revealed highly significant differences between the two simulated groups. There was also a significant increase in the number of edges (t_(48)_  = 3.19, p,  = 0.002, Cohen’s d  = 0.30) and in the clustering coefficient (t_(48)_  = 2.20, p,  = 0.03, Cohen’s d  = 0.21). In contrast, we found a significant decrease in the characteristic path length (t_(48)_ = −3.05. p  = 0.003, Cohen’s d  = 0.29).

Within the middle threshold range (0.50–0.54), there were no significant differences between the random networks of the two simulated groups in the first trial (10 thresholded connectivity matrices). However, for the second trial (25 thresholded connectivity matrices) there were only significant differences in the clustering coefficient (t_(23)_ = −2.19, p,  = 0.03, Cohen’s d  = 0.29) between the two simulated groups. The analysis of the number of edges displayed a trend to decreased number of edges in group one (t_(23)_ = −1.83, p,  = 0.07, Cohen’s d  = 0.23). In the third trial (50 thresholded connectivity matrices), the random network of the first group showed a decreased number of edges (t_(48)_ = −2.61, p  = 0.03, Cohen’s d  = 0.25) and a decreased clustering coefficient (t_(48)_ = −2.97, p  = 0.02, Cohen’s d  = 0.28) compared to the random network of the second group. The path length of the first group was significantly higher (t_(48)_  = 2.24, p  = 0.03, Cohen’s d  = 0.22).

**Figure 3:**
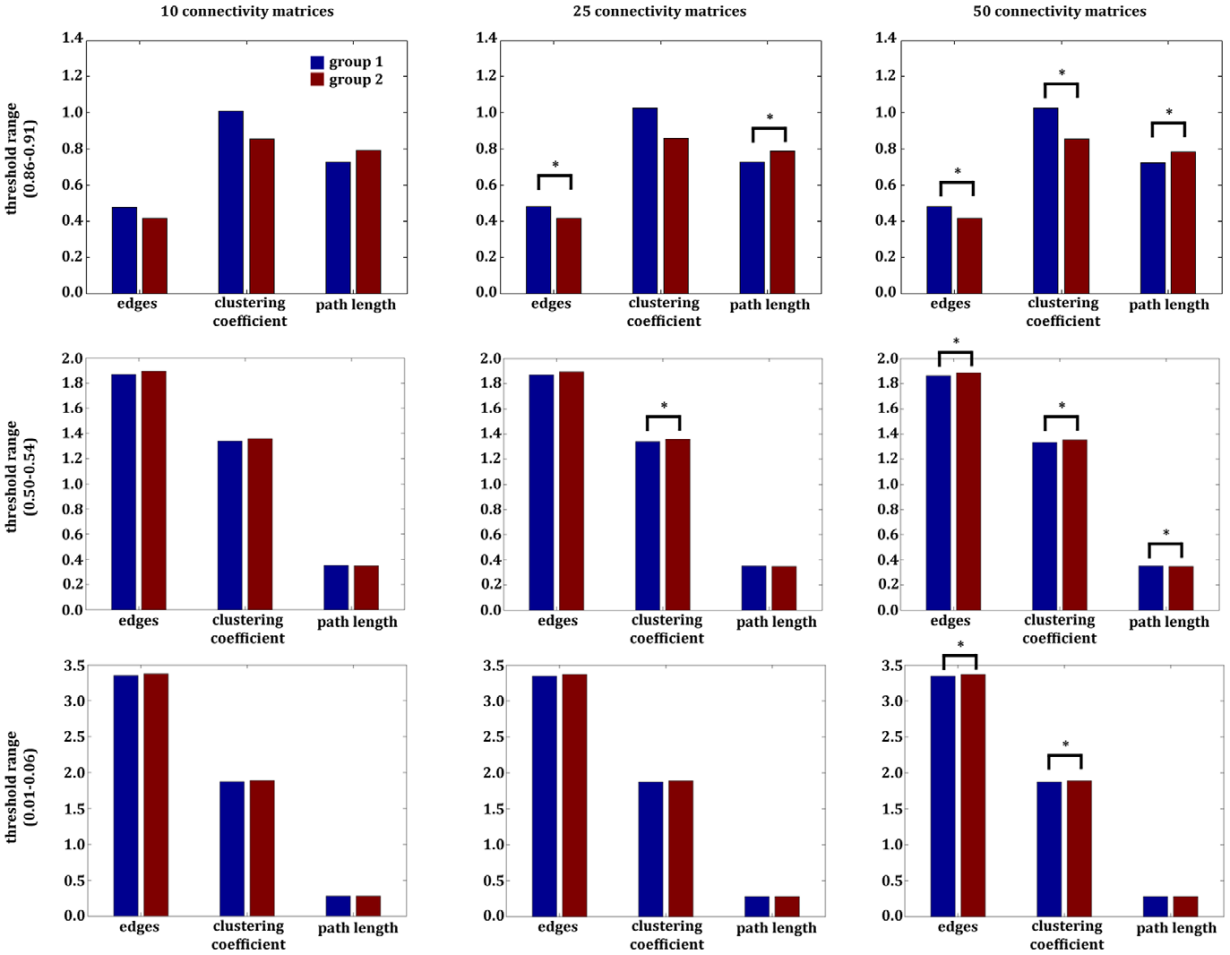
Results of the *multiple-thresholds-approach* of the example with the artificial data. Displayed are the results of the second example, which used artificial data. The comparison of the two networks, based on artificial data, revealed several significant differences. Depending on the number of thresholds (defining the different measurement units within each group) and the threshold range used for the comparison, completely distinct results could be obtained. For an optimized display, the numbers of edges were scaled (number of edges divided by 1000).

For the lower threshold range (0.001–0.06), the first and second trials revealed no significant differences, but the third trial showed (50 thresholded connectivity matrices) a lower number of edges (t_(48)_ = −2.41, p  = 0.02, Cohen’s d  = 0.23) and a lower clustering coefficient (t_(48)_ = −2.21, p  = 0.03, Cohen’s d  = 0.22) for the first group’s random network. All the results are presented in Figure 3.

### Group-level-permutation-statistics-approach

#### Example 1 - real data

The same data set was used as in the first example, which made use of multiple-thresholds-approach (see above). In line with the first example using the multiple-thresholds-approach, we created a mean connectivity matrix (averaged across all subjects), which was then thresholded with a set of different thresholds (range r  = 0.55–0.95, increments: 0.05). In the second step, small-world network parameters (clustering coefficient, path length) were calculated for the different thresholded mean coherence matrices. Here we present the results for the particular chosen threshold that best corresponds to a small-world topology (r  = 0.85). This threshold was applied to the mean connectivity matrices of the low and high IQ groups. This is only one of several possible approaches to choosing a threshold. In the upcoming discussion section we delineate the other possibilities. For more information regarding the results of the other thresholds please consider Table S1 and Figure S1.

As in the first example of the *multiple-thresholds-approach,* the subjects were allocated to a high or to a low IQ group based on a median-split, as previously described. The small-world network parameters were then calculated for the equally thresholded (threshold r  = 0.85) connectivity matrices of the low and the high IQ groups. The small-world network parameters of the high IQ group were then subtracted from the parameters of the low IQ group. In order to statistically test these differences, we used permutation statistics. Permutation tests are a sub-group of non-parametric statistics. The basic principle has originally been described by Fisher [26] and has been extended by others [27], [28], [29], [30]. The principle assumption is that within a test group all subjects are equivalent and that every subject is the same before sampling started [31]. From this point, one can compute a statistic and then observe the amount to which this statistic is distinctive by comparing the test statistics under rearrangements of the treatment assignments [26]. In contrast to classical parametric tests, which rely on theoretical probability distributions, permutation tests can be applied when the assumptions of parametric tests are untenable [31]. In situations where it is not feasible to compute the statistics for all the rearrangements, as is required in the Fisher’s exact test, a subsample can be used [27], [28]. Such a test is sometimes known as an approximate permutation test, because the permutation distribution is approximated by a subsample, also known as Monte-Carlo permutation tests or random permutation tests [31]. In the present study, we used the Edgington approach. To this end, we allocated the subjects randomly to one of two groups and created 1000 randomly assigned pairs of groups. For each random group pair, we calculated the mean correlation matrix and then the small-world parameters of the networks. In the second step of analysis, the differences in small-world network parameters between the pairs were obtained. To statistically prove the real differences between the high and the low IQ groups, we tested the real differences within the distribution of the randomly generated differences and a global level of significance was set at p<0.05. When setting the error probability to p<0.05, the real difference must exceed the extreme of 5% of the difference distribution, in order to reach statistical significance.

#### Results

**Figure 4:**
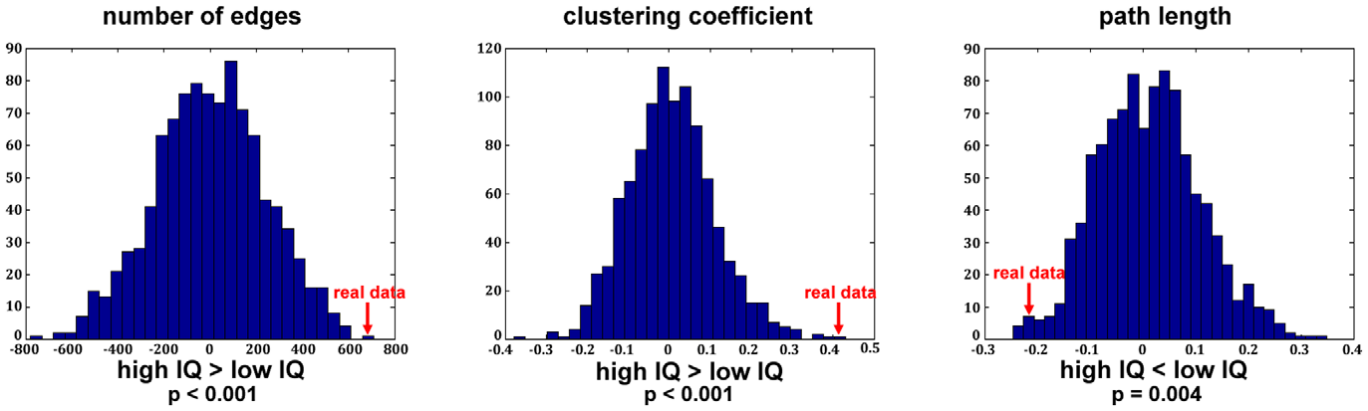
Results of the *group-level-permutation-statistics-approach* of the example with the real data. Displayed are the distributions of the randomly generated group pair differences. The red arrow indicates where the differences of the real data ( =  empirical difference between high and low IQ groups) are located within the distribution. The results show that the high IQ group revealed increased small-world network parameters.

The permutation analysis revealed that the high IQ group demonstrate significantly more edges than the low IQ group (p<0.001). Moreover, we found an increased clustering coefficient (p<0.001) and a decreased characteristic path length (p  = 0.004) for the high IQ group compared to the low IQ group. Thus, the high IQ group exhibits significantly more small-world topology. All results are summarized in Figure 4.

#### Example 2 - simulated data

In the present example, we used the same data set as in the second example of the multiple-thresholds-approach with artificial random networks (connectivity matrices). Again we assume to have two different groups with 30 subjects per group, but there is only one value per node and subject (i.e. cortical thickness or FA value in this specific region). We again have 84 simulated brain regions per subject, where we again allocated random values to each simulated brain region for each single subject. These data were used to construct the correlation matrix between all pairs of nodes, resulting in an 84×84 association matrix (network) for each group. They served as representation for the networks of two different groups.

However, instead of using the *multiple-thresholds-approach*, we now use no particular threshold, calculate the network parameters on the basis of the unthresholded data set, and subject these parameters to randomization tests, in order to conduct between-group comparisons. Since we did not threshold the connectivity matrices in this particular analysis, all connectivity matrices have an equal number of edges. Therefore, the between-group comparison of the number of edges is obsolete. Using unthresholded networks is only reasonable in the case of weighted networks (if every node is connected to every other node). Other alternative and valid approaches are discussed in the discussion section. However, the same procedure could also be applied to thresholded connectivity matrices. In the second step of analysis, we only computed the difference between the small-world parameters of the two groups. To statistically bolster this difference, we performed between-groups randomization tests by calculating different small-world parameters on the basis of 1000 randomized assignments of the subjects to the groups. We then computed a correlation matrix and small-world parameters for each randomization. This resulted in 1000 random group pairs. As for the originally assigned group, we again calculated the difference between the small-world parameters for each of the 1000 random group pairs, which resulted in 1000 difference values. These randomly achieved difference values now form the test-distribution and the difference of our originally assigned group of interest can now be tested using this distribution. A global level of significance was set at p  = 0.05. Since all groups were randomly generated, we assumed that there would be no significant differences regarding small-world topology.

#### Results

**Figure 5:**
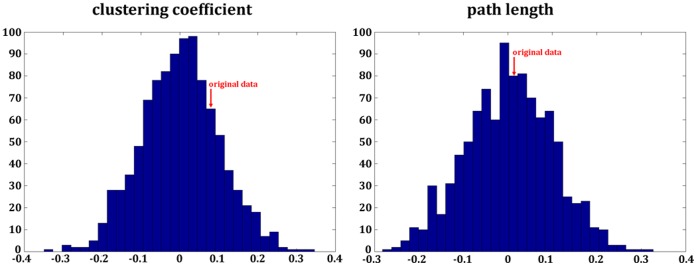
Results of the *group-level-permutation-statistics-approach* of the example with the artificial data. Displayed are the distributions of the randomly generated group pair differences. The red arrow indicates where the differences of the original data are located within the distribution. The results show, that there are no significant differences regarding the clustering coefficient or the characteristic path length.

The permutation analysis revealed no significant differences regarding the clustering coefficient (p  = 0.46, p>0.05) or the characteristic path length (p  = 0.88, p>0.05). The results are illustrated in Figure 5.

### Single-subject-connectivity-matrices-approach

#### Example 1–real data

The same data set was used as in Example 1 of the *multiple-thresholds-approach* and the *group-level-permutation-statistics-approach* (See above). In contrast to the two previous methods, we now used the correlation matrix of each subject instead of averaging the connectivity matrices over the entire group. The correlation matrices were thresholded by applying a set of different thresholds (r  = 0.65–0.95, increments: 0.05). The particular threshold, which identified the best small-world topology was chosen (r  = 0.85) and applied to the correlation matrices of each individual subject. For the results of the other thresholds, please refer to (Table S2). Obtaining single subject correlation matrices is only available for times series data (e.g. fMRI, EEG, MEG) or DTI with fibre tractography. Subsequently the correlation matrix of each subject was subjected to tnet software [21], [32], [33], which calculated the small-world indices for each individual subject (for further details see [19]). For statistical comparisons of the small-world networks, we compared the subjects of the low IQ group with those of the high IQ group (based on median-split in the RAPM performance) by calculating a t-test for independent samples. The global level of significance was set at p<0.05. Another possibility would be to calculate a regression analysis between the performance in the intelligence task and the small-world parameters, as was done in our previous study [19].

#### Results

The t-test for independent samples comparing the high IQ group vs. the low IQ group revealed a significantly increased number of edges (t_(57)_  = 2.83, p  = 0.006), a significantly increased clustering coefficient (t_(57)_  = 3.54, p  = 0.001), and a significantly decreased characteristic path length (t_(57)_ = −2.70, p  = 0.009) (See [19], for the results of the regression analysis).

#### Example 2–simulated data

In this example we used a similar data set as in the second example of the *multiple-thresholds-approach* and the *group-level-permutation-statistics-approach* with artificial random networks. Again, we assume to have two different groups with 30 subjects per group, but in this example we assume that each subject has an individual network, as is the case for MEG, EEG, resting fMRI, and DTI data when using tractography. We artificially created 60 networks with 84 nodes per network; representing for each subject a particular network. Subsequently the unthresholded weighted correlation matrix of each subject was subjected to tnet software, which calculated the small-world indices (clustering coefficient and characteristic path length) for each individual subject. The two groups were then compared with a t-test for independent samples (threshold was set p<0.05).

#### Results

The t-test for independent samples comparing the two groups did not reveal significant effects for the clustering coefficient (t_(58)_  = 0.24, p  = 0.81) or for the characteristic path length (t_(58)_  = 0.56, p  = 0.58).

## Discussion

Graph-theoretical approaches are an elegant way to describe functional or structural brain networks on the basis of large anatomical and neurophysiological data sets. Although attractive, these techniques are associated with some statistical problems, which have been described in this paper. A major problem is on which basis inferential statistics are performed when statistically testing the measures obtained from graph-theoretical analyses. A typical approach is to compare the graph-theoretical measures between two different groups. Several papers have adopted the multiple-thresholds-approach by using different thresholds for which different graphs are computed separately for each group. The obtained graph-theoretical measures for each group are then subjected to between-groups statistical test. Typically this approach is used in the context of graph-theoretical network analyses conducted with cortical thickness and FA data. Since for each voxel or region there is only one data point available, connectivity measures can only be computed for an entire group. Thus, there is no distribution of measures available to calculate statistical tests. To generate the necessary distribution of measures for classical inferential statistics, some studies generated an artificial distribution by thresholding the networks on group level over a range of thresholds and thus collected several connectivity measures. These different measures were then subjected to between-groups statistical tests. One problem with this approach is that these measures are not independent from each other since information of denser networks (thresholded using low thresholds) is also included in sparser networks (thresholded using high thresholds). These networks and thus the derived measures are strongly inter-correlated and should not be treated as coming from different subjects. This is a serious problem, especially for parametric inferential statistical analyses, which requires independence between the measurements. A further problem is that the power of the statistical tests strongly depends on the number of measurements and in this case on the number of thresholds used.

We demonstrated these problems on the basis of a real EEG data set and simulated data. As expected the p-values strongly depend on the number of thresholds. Thus, a researcher could easily manipulate the obtained p-value by arbitrarily manipulating the number of thresholds until he/she obtained the p-value she/he would like to achieve. In order to circumvent this problem effect size measures are more suitable because they are independent from sample size. In fact, we demonstrated similar effect size measurements that were independent of the number of thresholds. Therefore, effect sizes are an important measurement, which should be added to the p-values if one still uses the multiple-thresholds-approach. If one is really interested in comparing the profiles of the network parameters across the different thresholds, randomization tests should be used since they do not need independence of the data.

We described two different approaches, which in a valid manner can indeed deal with the non-independency problem, namely, the group-level-permutation-statistic-approach and the single-subject-connectivity-matrices-approach. For intra-subject connectivity measures, like correlations between time series of resting-state fMRI, coherence measures of EEG or measures dependency obtained by fibre tracking in diffusion tensor imaging both suggested approaches are applicable. Whether the group-level-permutation-statistic-approach or the single-subject-connectivity-matrices-approach should be employed depends on the available data and the deployed research question. The advantages of the randomisation procedure are that permutation statistics can be applied when the assumptions of classical inferential statistics are untenable or distribution of the data is unknown and sample size is small [31]. An additional advantage is that an exact p-value (or a marginally exact p-value when Monte-Carlo procedure is used) can be calculated. The disadvantages of permutation tests are that the computation time could be very extensive, and that they also tend to be conservative. Further advantages and disadvantages could be found in [34] and [31]. Nevertheless, the group-level-permutations-statistics-approach is to our knowledge the only valid approach for using connectivity measures obtained on the basis of inter-subject correlations (i.e. structural MRI and DTI, when only using FA-values). The advantage of the single-subject-connectivity-matrices-approach is that it permits the use of single subject variance for statistical analysis (e.g. regression analysis). The disadvantage of this approach is that it is only available for intra-subject data (e.g., times-series data in fMRI, MEG or EEG, as well as DTI, when fibre tracking is used).

Another unsolved problem within the tresholding procedures is that there is currently no definitive and generally accepted strategy for applying particular thresholds in graph-theoretical networks analyses. How large should the threshold steps be? What are the smallest or largest thresholds that one can use? There are currently no concrete answers to these questions. Nonetheless, we present here and in our previous studies [13], [19], [35] several possibilities to proceed if the connectivity measures are obtained by means of group level dependency. Probably the best way to circumvent the problem is to threshold the connectivity matrix over a wide range of thresholds and to then conduct the permutation analysis for each threshold individually as described above and in Tables S1 and S2. However, one has to face a problem with these approaches, which is the tremendous computation time for these analyses. For example, to perform a randomization test as described in the context of our single-subject-connectivity-matrices-approach with 84 nodes six days of computation time is needed for a standard workstation. When using more nodes, computation time exponentially increases to weeks or even months for the same workstation.

Using unthresholded weighted connectivity matrices (as it was demonstrated above) is another possibility to statistically test the network parameters, but this approach can also generate long computation times. In addition, thresholded networks exhibit a clearer small-world topology, because the noise of the data is reduced by the thresholding procedure [2]. In our previous study, we presented a further possibility [13], . We first determined the threshold, which exhibited the best small-world topology and then used this threshold. This is only one of several possibilities to choose a particular threshold. Most studies use thresholds over a predefined range that are defined a priori. This approach is adequate if these differently thresholded matrices are not used as independent measures or for parametric statistical tests.

Using graph-theoretical network approaches in the context of neuroscience research is a relatively young scientific field. Although promising this approach can be associated with some methodological problems. Apart from the thresholding problem there are several further methodological issues. For example the set of nodes of the network has to be carefully selected and determines largely the connection and therefore also the interpretation of the brain networks [36], [37]. For the instance of interpretability, nodes should represent brain regions and are supposed to be inherently independent from other nodes. The relationship between two nodes is not meaningful when the nodes are too similar to each other. Let’s imagine spatially smoothed voxels sharing similar information because the spatial smoothing filter induces similar information in these adjacently located voxels. Thus, the spatial smoothing induces a kind of artificial correlation between neighboring voxels and can mask physiological similarity or dissimilarity. On the other hand parcellation schemes that link heterogeneous brain regions into a single node might be meaningless as well.

For most studies anatomical templates as Brodmann areas or the Automated Anatomical Labeling (AAL) atlas were used. An immense advantage of using an anatomical template is that different networks of different studies, even functional and structural networks, could be directly compared. So fMRI, structural MRI, and DTI data most often use one of these template maps. The disadvantage of this template maps is that the regions can vary extremely in the size (number of voxel within a nodes). Therefore, new approaches have been developed to define nodes. One promising approach is to define the nodes on the basis of data-driven techniques [38]. Most studies defined the electrodes as nodes for brain graphs based on data obtained with microelectrodes on cortical tissue or surface sensors in MEG and EEG [39]. This can cause strong correlations between neighboring electrodes due to volume conduction of electrical activity from a single source to multiply nearby electrodes on the scalp surface, which can confound the results of the graph-theoretical analysis [40]. A better approach is to reconstruct the sources and define each source as a node [19], [41], [42], [43], [44]. In practice there is no unique answer to legitimate the choice of connection between nodes (edges) and they are highly dependent on the conditions of acquisition and preprocessing. There is an extensive literature about measuring the connectivity in fMRI nicely reviewed by Smith et al. [45]. Thus, graph theoretical analysis of neuroimaging data is not a simple “plug and play“ application. It is rather a model-based approach, demanding arbitrary assumptions and decisions, which can have significant effects on the outcomes of the analysis. Moreover, there is no best way how to compare topological metrics between graphs and it is in general not a trivial question to solve. In addition to these relatively specific issues about construction and comparison of brain networks, any procedure to conduct graph theoretical analysis of neuroimaging data also put up a number of inquires about data acquisition, preprocessing, statistical tests, multiple comparisons and visualization.

Taken together there are several valid possibilities of dealing with thresholding in network analysis. The choice of the applied approach should be decided based on the particular hypothesis, the amount of data, the methods used for network analysis, and the resources that are available for the computations. We suggest that if there is the possibility to calculate a connectivity matrix for each individual subject, then one should not create mean connectivity matrices for a whole group and compare this mean connectivity between different groups.

## Supporting Information

Figure S1 Displayed are the distributions of the randomly generated group pair differences for all thresholds. The red arrow indicates where the differences of the real EEG data are located within the distribution. The results of all thresholds show, that the high IQ group revealed increases small-worldness.
(DOC)

Table S1 Listed are the p-values for each small-world parameter of the permutation statistics of the first example (EEG data) of all thresholds. We compared the difference of the real EEG data to 1000 randomly generated group pairs. All threshold showed an increased small-worldness for the high IQ group.
(DOC)

Table S2 Listed are the t-values and p-values for each small-world parameter of the single subject method of the first example (EEG data) for all thresholds. We compared the small-world parameters between the high and the low IQ group for each threshold separately. All threshold showed an increased small-worldness for the high IQ group.
(DOCX)
