# Supplementary figures and images for: The Problem of Thresholding in Small-World Network Analysis
Source: PLoS One. 2013 Jan 3;8(1):e53199. doi: 10.1371/journal.pone.0053199 (PMC3536769; doi:10.1371/journal.pone.0053199)

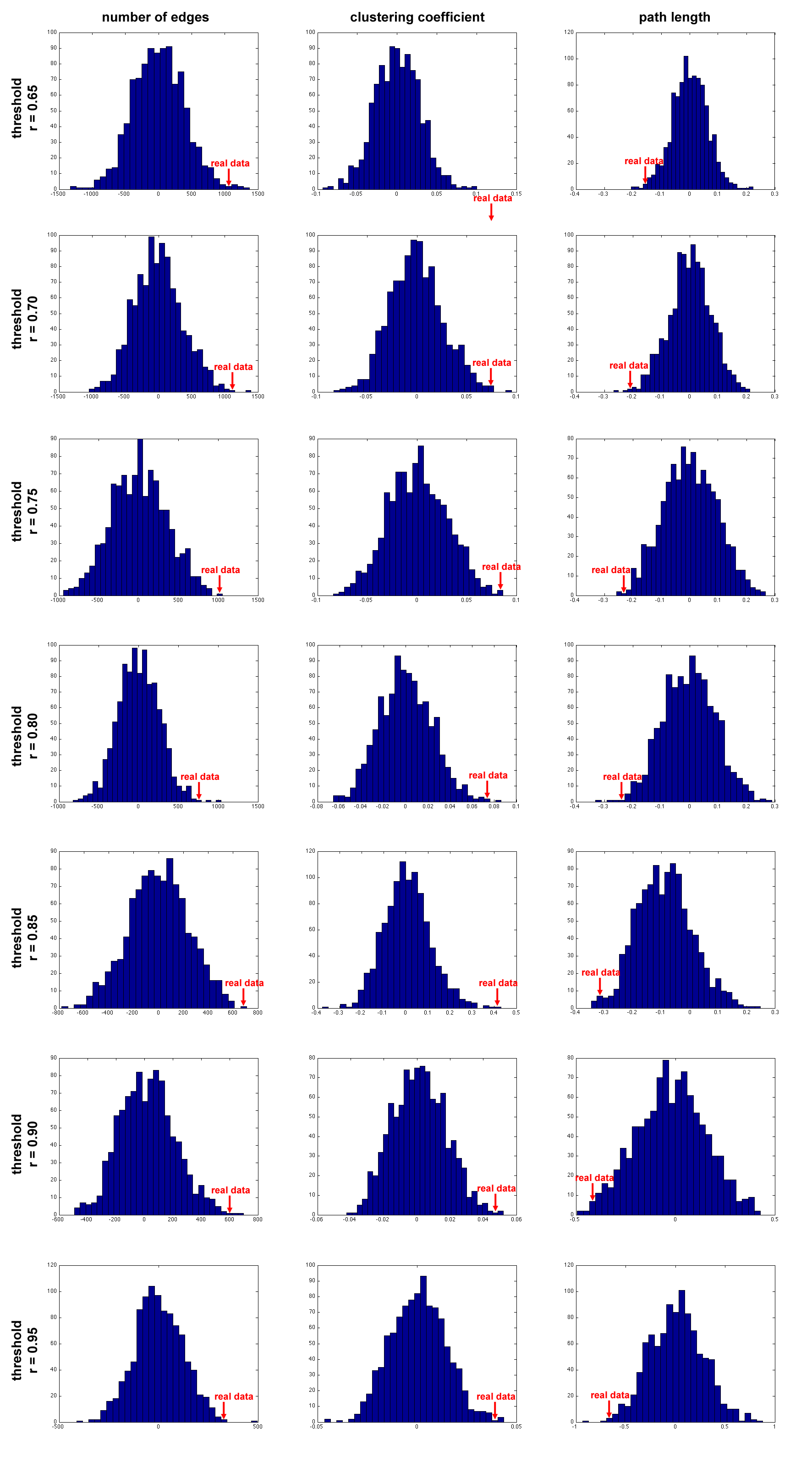

Supplement: Figure S1 — Displayed are the distributions of the randomly generated group pair differences for all thresholds. The red arrow indicates where the differences of the real EEG data are located within the distribution. The results of all thresholds show, that the high IQ group revealed increases small-worldness. (DOC) [file pone.0053199.s001.doc]

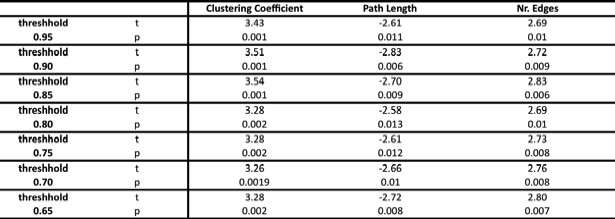

Supplement: Table S2 — Listed are the t-values and p-values for each small-world parameter of the single subject method of the first example (EEG data) for all thresholds. We compared the small-world parameters between the high and the low IQ group for each threshold separately. All threshold showed an increased small-worldness for the high IQ group. (DOCX) [file pone.0053199.s003.docx]
