# Supplementary material for: The Problem of Thresholding in Small-World Network Analysis
Source: PLoS One. 2013 Jan 3;8(1):e53199. doi: 10.1371/journal.pone.0053199 (PMC3536769; doi:10.1371/journal.pone.0053199)
Supplement: Supplementary file 2 [file pone.0053199.s002.doc]

		Nr. Edges	Clustering Coefficient	Path Length
**threshhold 0.95**	p	0.001	0.003	0.003
**threshhold 0.90**	p	0.003	0.003	0.004
**threshhold 0.85**	p	<0.001	<0.001	0.004
**threshhold 0.80**	p	0.002	0.001	0.003
**threshhold 0.75**	p	0.001	<0.001	0.003
**threshhold 0.70**	p	0.002	0.001	0.004
**threshhold 0.65**	p	0.006	0.001	0.007
